# The *Streptococcus agalactiae* complement interfering protein combines multiple complement-inhibitory mechanisms by interacting with both C4 and C3 ligands

**DOI:** 10.1096/fj.201801991R

**Published:** 2018-12-19

**Authors:** Stefania Giussani, Giampiero Pietrocola, Danilo Donnarumma, Nathalie Norais, Pietro Speziale, Monica Fabbrini, Immaculada Margarit

**Affiliations:** * GlaxoSmithKline (GSK), Siena, Italy; and; † Unit of Biochemistry, Molecular Medicine Department, University of Pavia, Pavia, Italy

**Keywords:** GBS, neonatal infection, B cells, virulence factor

## Abstract

Group B *Streptococcus* (GBS) colonizes the human lower intestinal and genital tracts and constitutes a major threat to neonates from pregnant carrier mothers and to adults with underlying morbidity. The pathogen expresses cell-surface virulence factors that enable cell adhesion and penetration and that counteract innate and adaptive immune responses. Among these, the complement interfering protein (CIP) was recently described for its capacity to interact with the human C4b ligand and to interfere with the classical- and lectin-complement pathways. In the present study, we provide evidence that CIP can also interact with C3, C3b, and C3d. Immunoassay-based competition experiments showed that binding of CIP to C3d interferes with the interaction between C3d and the complement receptor 2/cluster of differentiation 21 (CR2/CD21) receptor on B cells. By B-cell intracellular signaling assays, CIP was confirmed to down-regulate CR2/CD21-dependent B-cell activation. The CIP domain involved in C3d binding was mapped *via* hydrogen deuterium exchange–mass spectrometry. The data obtained reveal a new role for this GBS polypeptide at the interface between the innate and adaptive immune responses, adding a new member to the growing list of virulence factors secreted by gram-positive pathogens that incorporate multiple immunomodulatory functions.—Giussani, S., Pietrocola, G., Donnarumma, D., Norais, N., Speziale, P., Fabbrini, M., Margarit, I. The *Streptococcus agalactiae* complement interfering protein combines multiple complement-inhibitory mechanisms by interacting with both C4 and C3 ligands.

*Streptococcus agalactiae* [group B *Streptococcus* (GBS)] is a gram-positive commensal microorganism normally present in the human gastrointestinal and genitourinary tract. GBS is also an opportunistic pathogen that can cause life-threatening sepsis and meningitis in infants, systemic infections in the elderly, and bovine mastitis (1–3). Peripartum antibiotic prophylaxis has reduced infections occurring in the first week of life but has no effect on late-onset disease, and a vaccine is not yet available (4).

GBS is endowed with a number of virulence factors that facilitate interactions with multiple targets to penetrate host barriers and to counteract innate immune responses. Complement effectors serve as first line of defense against GBS infection, promoting phagocytic killing of these bacteria by neutrophils and macrophages (5). Therefore, the pathogen has evolved several mechanisms to inhibit complement activation to colonize and invade its host. GBS is surrounded by a thick, polysaccharidic capsule, containing sialic acid (6–8), which masks the bacterial surface and interferes with complement deposition (9). Antibodies directed to each of the 10 existing polysaccharide variants can block that effect and mediate bacterial serotype-specific phagocytic killing, making the GBS capsule a promising vaccine target. In addition to the capsular shield, GBS expresses cell wall–anchored proteins that manipulate complement attack by binding host regulators or by degrading its effectors, which have also been described as potential protective vaccine candidates. Among these are the Alp family protein members (6, 10) and the streptococcal histidine triad (SHT) protein (11) that bind factor H to accelerate the decay of the alternative pathway C3 convertase, the BibA protein that interacts with the classical and lectin pathway regulator C4 binding protein (12) and ScpB, which exerts a proteolytic effect on the anaphylatoxin C5a (13, 14).

In a recent study focusing on GBS-secreted virulence factors that could potentially interfere with human complement, we identified a 153-residues polypeptide that was named complement interfering protein (CIP) (15). The protein showed partial homology to the well-studied *Staphylococcus aureus* complement modulator extracellular adherence protein (Eap) (16, 17). Similar to Eap, the secreted CIP binds to the bacterial surface *via* an unknown ligand and interacts with C4 and C4b complement effectors. CIP was shown to prevent the formation of the C4b2a convertase, a common step in the classical- and lectin-complement pathways, to modulate the formation/deposition of C3b on the GBS surface and to prevent phagocytic killing of the microorganism.

CIP also shows partial homology to the *Staphylococcus aureus*–secreted proteins extracellular fibrinogen-binding protein Efb (18), extracellular fibrinogen-binding homologous protein (Ehp) (19), and staphylococcal-binding IgG (Sbi) (20, 21) (10–13% identity and 15–20% similarity) (15). These 3 proteins are incapable of binding C4 ligands, but can interact with the C3 central component of complement and its fragments C3b, iC3b, C3dg, and C3d (18–23). Functional and structural studies have revealed that binding of Efb, Ehp, and Sbi to C3b induces conformational changes in this complement effector, which blocks its interaction with factor B, thereby impairing the formation of the alternative pathway C3 convertase C3bBb complex (16, 19, 23). Further, binding of Efb and Ehp to the C3d ligand can affect the interaction of this molecule with the complement receptor 2/cluster of differentiation 21 (CR2/CD21) member of the B-cell surface coreceptor complex implicated in the maturation of B cells when encountering specific antigens (22, 24).

The present study was aimed at investigating the binding capacity of CIP toward C3 and its derived C3b and C3d fragments by biophysical methods and its ability to interfere with B-cell activation by inhibiting the interaction between the C3d complement ligand and its CR2/CD21 receptor.

## MATERIALS AND METHODS

### Cell cultures and media

Raji cells (American Type Culture Collection, Manassas, VA, USA) were cultured in RPMI-1640 (GlutaMax; Thermo Fisher Scientific, Waltham, MA, USA) supplemented with 10% fetal calf serum (GE Healthcare, Waukesha, WI, USA) and Pen/Strep (MilliporeSigma, Burlington, MA, USA). B cells were isolated from human peripheral blood mononuclear cells (PBMCs; MAT Biotech, Leiden, The Netherlands) using the Human B Cell Purification Kit II, following manufacturer’s instructions (Miltenyi Biotec, Bergisch Gladbach, Germany).

### Far-Western blot and ELISA analysis of CIP interaction with complement factors

Affinity-purified, His-tagged CIP was subjected to 12.5% SDS-PAGE (5 μg/lane), electroblotted onto a nitrocellulose membrane, and incubated with 1% (v/v) normal or C3-depleted human serum (MilliporeSigma). The membrane was probed with goat anti-C3 serum (Complement Technology, Tyler, TX, USA) and horseradish peroxidase (HRP)-conjugated mouse anti-goat antibodies (Agilent Technologies, Santa Clara, CA, USA).

C3 and its C3b and C3d fragments (Merck, Darmstadt, Germany) were loaded onto a 12.5% SDS-PAGE gel and electroblotted. The membrane was overlaid with CIP diluted to 1 μg/ml in PBS-milk 3%–Tween 20 (0.05%), followed by primary rabbit anti-CIP antiserum and then goat HRP-conjugated anti-rabbit antibodies (1:1000).

Nunc MaxiSorp microtiter plates (Thermo Fisher Scientific) were coated with 100 ng of C3, C3b, C3d, C1q, and factor I and incubated overnight at 4°C in 50-mM carbonate buffer (pH 9.5). The wells were washed 2 times with PBS supplemented with 0.1% (v/v) Tween 20 (PBST), blocked with 2% (w/v) bovine serum albumin (BSA) in PBST for 1 h at 22°C, and probed with increasing concentrations of CIP, followed by incubation with rabbit anti-CIP antibody (1:1000), and then with a HRP-conjugated goat anti-rabbit IgG (1:1000). The signal was revealed by HRP enzymatic activity. To calculate the relative affinity association constant (*K*_a_) of CIP for C3, C3b, and C3d human ligands, the data were fitted using the following equation: *A* = *A*_max_ [*L*]*K*_a_/(1 + *K*_a_[*L*]), where [*L*] is the molar concentration of the ligand. The reported dissociation constants (*K*_d_) were calculated as reciprocals of the *K*_a_ values. The assays were performed ≥2 times for each protein, and the *K*_d_ values obtained were reproducible in all cases.

In competitive ELISA experiments with immobilized C3b and C4b, Nunc MaxiSorp Microtiter plates were incubated overnight at 4°C with 100 ng of each protein in 50 mM carbonate buffer (pH 9.5). The plates were washed as previously described and probed with 0.6 µM CIP preincubated or not with equimolar amounts of C3b or C4b. The binding was revealed with rabbit anti-CIP antibody (1:1000), followed by a HRP-conjugated goat anti-rabbit IgG (1:1000).

For ELISA detection of C3d/CIP and C3d/CD21 complexes under increasing ionic strength conditions, 250 ng of C3d were surface coated onto microtiter plates, and CIP or CD21 were diluted in a buffer containing increasing NaCl concentrations and added to the wells. The complex formation was revealed through rabbit anti-CIP or anti-CD21 serum (1:1000) and goat anti-rabbit HRP.

### Surface plasmon resonance analysis of CIP–C3d interaction

The affinity of the interaction between CIP and C3d was evaluated with a BIAcore X100 instrument (GE Healthcare). Human C3d was covalently immobilized on a dextran matrix CM5 sensor chip surface with a C3d solution (30 μg/ml in 50 mM sodium acetate buffer, pH 5) diluted 1:1 with *N*-hydroxysuccinimide and *N*‑ethyl‑*N*′‑(3‑dimethylaminopropyl)carbodiimide hydrochloride. The excess of active groups on the dextran matrix was blocked with 1 M ethanolamine (pH 8.5). On another flow cell, the dextran matrix was treated as previously described but without any ligand to provide an uncoated reference flow cell. The running buffer used was PBS containing 0.005% (v/v) Tween 20. A 2-fold linear dilution series (5 μM up to 0.0390 μM) of CIP in running buffer were passed over the ligand at the flow rate of 45 ml/min, and all the sensorgrams were recorded at 22°C. Surface regeneration was achieved by injecting a solution of 25 mM NaOH. Association and dissociation kinetics parameters (*K*_a_ and *K*_d_) and the equilibrium dissociation constant *K*_d_ were estimated with a 1:1 interaction model (Langmuir model) by nonlinear fitting, using BIAevaluation 1.0 software (GE Healthcare).

### Competitive ELISA

Microtiter wells were coated with 100 ng of soluble CR2/CD21 (Sino Biological, Beijing, China) and incubated overnight at 4°C in 50 mM carbonate buffer (pH 9.5). The wells were washed 3 times with PBST, blocked with 2% (w/v) BSA in PBST for 1 h at 22°C, and then probed with C3d (100 ng/well) preincubated with serial dilutions of CIP in PBS, followed by incubation with anti-C3 goat pAb (1:2000; Complement Technology) and then HRP-conjugated rabbit anti-goat IgG (1:1000; Agilent Technologies). Binding of the secondary antibody was revealed by HRP enzymatic activity.

### Flow cytometry analysis of C3d interaction with CR2/CD21 on B cells

Biotinylated C3d (bC3d) was prepared using a Biotin Type A Conjugation Kit (Abcam, Cambridge, MA, USA), dialyzed to eliminate the excess of free biotin and incubated with streptavidin (SA; MilliporeSigma) for 30 min at 37°C. The bC3d–SA complex was subsequently incubated for 30 min at 37°C with His-tag–purified CIP at 1, 3, 6, and 9 μM to obtain CIP–bC3d–SA. The GBS-secreted, recombinant fibrinogen-binding protein 3 (Fib3) was used as a negative control at 9 μM (25). The bC3d–SA or CIP–bC3d–SA or Fib–bC3d–SA complexes were then added to Raji cells or B cells purified from human PBMCs (5 × 10^5^/sample), and the mixture was left 15 min at 37°C and washed with PBS. Bound C3d was then stained with a mouse anti-C3d mAb (prediluted 1:100; Thermo Fisher Scientific) and revealed with a phycoerythrin-labeled anti-mouse secondary antibody (Jackson ImmunoResearch Laboratories, West Grove, PA, USA). Both incubations were conducted for 10 min at 37°C, and after each incubation, 2 washes in PBS–fetal calf serum 1% (v/v) were performed. Cells were fixed with Cytofix (BD, Franklin Lakes, NJ, USA) for 20 min at 4°C and analyzed using a Canto II Flow Cytometer with FACSDiva Software (BD). The geometric mean of the peaks was calculated through the FlowJo software (Tree Star, Ashland, OR, USA). The signals obtained from the samples with no CIP added were considered as 100% of C3d bound to CR2/CD21 on Raji cells and were used to normalize the signals obtained from the CIP-containing complex. The graph shows the residual C3d binding derived from the mean fluorescence intensity values of the peaks analyzed with the FlowJo software.

### Calcium mobilization on Raji cells

Raji B cells (1 × 10^6^/ml) were incubated with Fura Red (Molecular Probes, Thermo Fisher Scientific) in PBS with 2 mM EDTA at 37°C for 40 min and washed twice with the same buffer. The stimulus was formed by coincubating 0.1 μM C3d-biotin, 1 μM SA, and 0.2 μM biotinylated anti-human-IgM for 30 min at 37°C. To assess the inhibition of intracellular signaling by CIP, increasing CIP concentrations (0.5, −5, and −20 μM) were preincubated with biotinylated C3d before stimulus formation. Fib3 (20 μM) was used as negative control. Fluorescence detection was performed on 500 μl of cells (5 × 10^5^/sample) using a LSRII Flow Cytometer (BD Biosciences). After establishing a baseline for 60 s, Raji cells were stimulated by the addition of the preformed stimuli (100 μl). Changes in Fura Red fluorescence emission were recorded using the LSRII. Data were analyzed through the FlowJo software and plotted as function of time and Fura Red emission ratio.

### Hydrogen deuterium exchange-mass spectrometry

Sample preparation, digestion, and separation for hydrogen deuterium exchange-mass spectrometry (HDx-MS) analysis were performed as previously described (26). Briefly, the CIP–C3d complex was formed with 45 pmol of both CIP and C3d and incubated for 30 min at room temperature and then for 10 min on ice. The deuteration was initiated by diluting the sample with deuterated PBS and performed on ice. At different times during deuteration, samples were removed for quenching and dissociation of the protein–protein complex and immediately frozen in liquid nitrogen. A control experiment without C3d was performed using the same conditions. Labeled samples were rapidly thawed to 0°C and injected into a nanoAcquity ultraperformance liquid chromatographic system with HDX technology (Waters, Milford, MA, USA). Samples were digested online with a Poroszyme Immobilized Pepsin Cartridge (Thermo Fisher Scientific), and the generated peptides were trapped, concentrated, desalted, and separated on a reverse-phase Acquity Ultraperformance Liquid Chromatography Ethylene Bridged Hybrid C18, 1.7 μm, 1.0 × 100-mm column (Waters). Mass spectra were acquired in resolution mode (*m*/*z* 100–2000) on a Waters Synapt-G2 mass spectrometer equipped with a standard electrospray ionization source. The identity of each peptide was confirmed by mass spectrometry elevated energy, as previously reported (26). Data were processed with a Protein Lynx Global Server 2.5 (Waters), and each fragmentation spectrum was inspected manually to confirm the assignment. The DynamX software (Waters) was used to select the peptides considered for the analysis and to extract the centroid mass of each of them and for each charge state as a function of the labeling time. Only the peptides present in ≥4 repeated digestions of the unlabeled proteins were considered for the analysis.

### Sequence alignment and statistical analysis

Sequences of Efb-C and Sbi were downloaded from the UniProt Knowledgebase (UniProt Consortium, Munich, Germany), and sequence alignment was performed with the Geneious software (Biomatters, Auckland, New Zealand) setting BLOSUM62 Matrix to calculate sequence identities and similarities.

Statistical analysis was performed with Prism software (GraphPad, La Jolla, CA, USA).

## RESULTS

### Interaction of CIP with C3 and its C3b and C3d fragments

**Figure 1:**
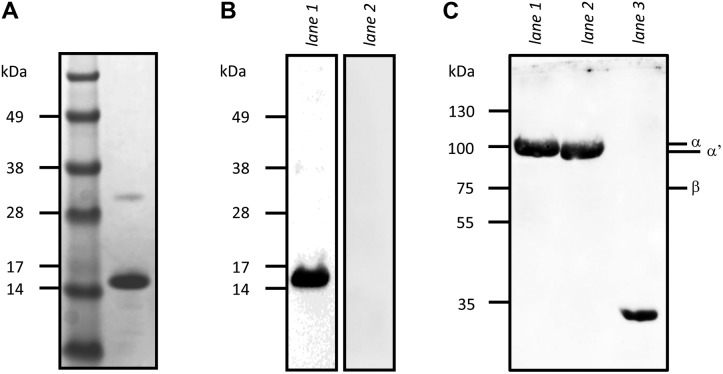
Far-Western blot analysis revealing the interaction between CIP and C3, C3b, C3d. *A*) SDS-PAGE analysis of affinity-purified, His-tagged CIP used in this study. *B*) CIP was subjected to SDS-PAGE, electroblotted onto a nitrocellulose membrane, and incubated either with normal (lane 1) or C3-depleted (lane 2) human serum; the membrane was probed with anti-C3 serum and HRP-conjugated secondary antibody. *C*) C3, C3b, and C3d (lanes 1–3, respectively) were loaded onto SDS-PAGE, and the electroblotted membrane was overlaid with CIP, followed by a primary anti-CIP serum and then an HRP-conjugated secondary antibody to reveal the CIP binding. The expected MW of C3α, C3bα′, and C3β are indicated on the right.

We first investigated whether CIP showed a binding capacity toward C3 present in human serum by Far-Western blot assays. A His-tagged, recombinant form of CIP was highly purified from *Escherichia coli*, analyzed by SDS-PAGE (**Fig. 1*A***), and electroblotted onto nitrocellulose membranes. The membranes were incubated with normal or C3-depleted human serum before immunostaining with an anti-C3 pAb (Fig. 1*B*, lanes 1 and 2, respectively). A single protein band with an apparent MW of 15 kDa in agreement with the monomeric form of CIP was revealed on the membrane incubated with normal serum, whereas no signal was detected in the absence of C3, evidencing a specific interaction between CIP and C3.

In a second set of experiments, purified C3 and its C3b and C3d fragments were separated by electrophoresis under reducing conditions, electroblotted onto a membrane that was incubated with soluble, recombinant CIP, and then immune-stained with a CIP pAb (Fig. 1*C*, lanes 1–3). The data confirmed that the CIP protein interacted with the α chain of C3 (expected MW 110 kDa) and revealed an interaction with the α′ chain of its active form C3b (101 kDa) and with the C3d (35 kDa) degradation fragment, whereas no staining was detected for the band corresponding to the MW of the C3 and C3b β chain (75 kDa).

**Figure 2:**
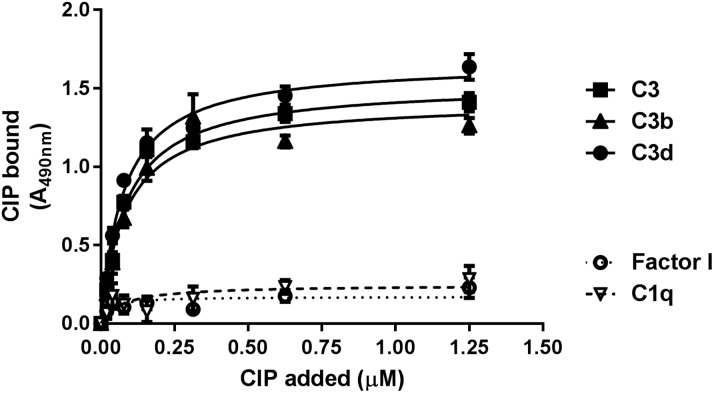
Dose-dependent binding of CIP to surface-coated C3, C3b, and C3d. Microtiter wells were coated with 100 ng of complement factors and incubated with increasing concentrations of CIP; bound protein was detected by anti-CIP polyclonal serum, followed by an HRP-conjugated secondary antibody.

A dose-dependent and saturable interaction between CIP and C3, C3b, or C3d ligands was confirmed by ELISA. Surface-coated C3, C3b, and C3d were overlaid with increasing concentrations of purified CIP, which was followed by incubation with anti-CIP mouse serum and an HRP-conjugated secondary antibody (**Fig. 2**). Apparent *K*_d_ values (means ± sd) of 84.83 ± 0.009, 82.42 ± 0.0157, and 75.96 ± 0.0084 nM were estimated for the binding of CIP with C3, C3b, and C3d, respectively. These affinity values were comparable to those previously obtained for the interaction between CIP and C4b (15).

**Figure 3:**
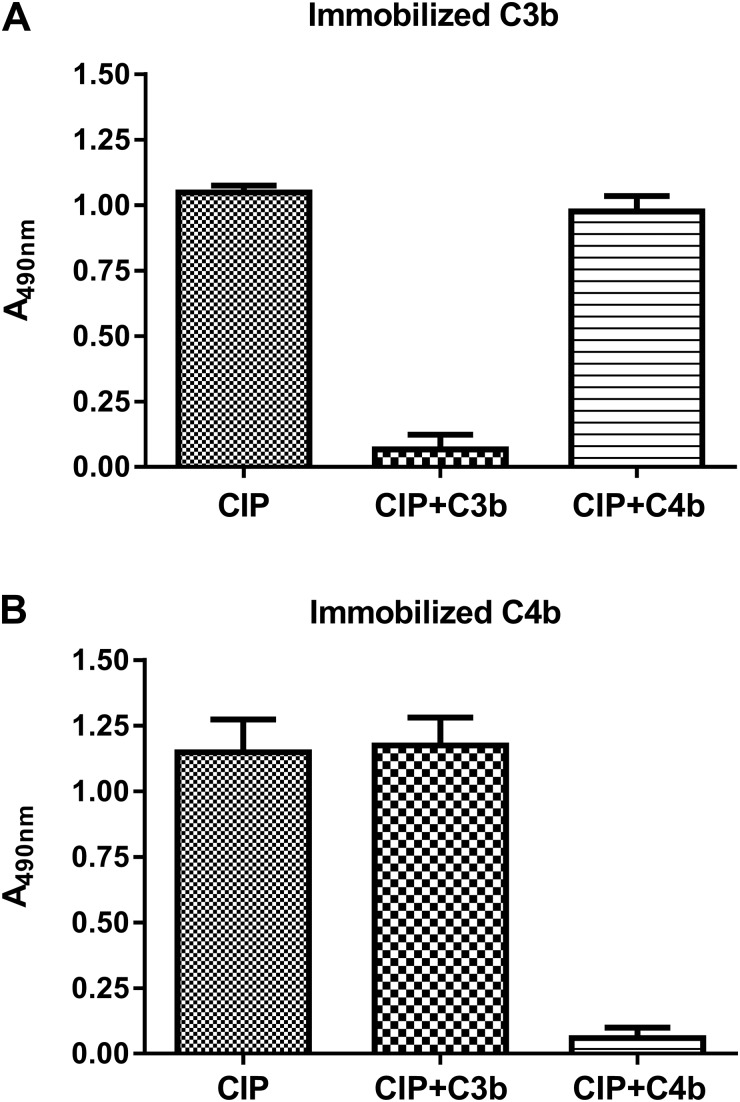
Competitive inhibition assessment of CIP interaction with C3b and C4b. *A*) Results of an experiment with 100 ng of C3b was coated on a 96-well plate and overlaid with 0.6 µM of CIP preincubated with equimolar amounts of C3b or C4b, followed by incubation with anti-CIP IgG pAb and then an anti-mouse HRP-conjugated antibody. *B*) A similar experiment in which 100 ng of C4b was immobilized and overlaid with 0.6 µM of CIP preincubated with equimolar amounts of C4b or C3b.

We sought to investigate by competitive ELISA whether CIP binding to its C4b and C3b ligands could occur at different sites on the GBS protein. Surface-coated C3b or C4b was overlaid with purified CIP in the absence or presence of equimolar concentrations of soluble C4b or C3b, washed with PBST, and incubated with anti-CIP mouse serum and HRP-conjugated secondary antibody. As shown in **Fig. 3**, soluble C3b inhibited binding of CIP to immobilized C3b, but not to C4b. Further, soluble C4b inhibited binding of CIP to immobilized C4b, but not to C3b. The above data suggested that the double-binding capacity of CIP toward C4 and C3 ligands relied on independent interaction sites.

### Biochemical characterization of the interaction between CIP and C3d

**Figure 4:**
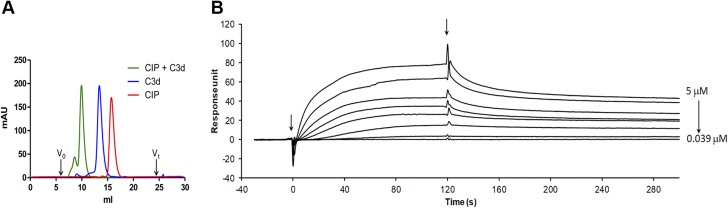
Analysis of the interaction between CIP and C3d by size-exclusion chromatography and SPR. *A*) CIP and C3d were loaded separately or mixed in an equimolar concentration onto a Superdex column; the MW of eluted peaks was determined from a calibration curve. *B*) Two-fold linear dilution series (0.0390–5 μM) of CIP was injected over the C3d surface (250 response units) of a CM5 sensor chip. The sensorgrams obtained were normalized *vs.* the response obtained when the recombinant GBS protein was flowed over uncoated chips. Each sensorgram was evaluated with BIA 3.0 software provided with the system. Shown is 1 representative of 3 experiments in which the start and the end of injection are indicated by arrows.

The stoichiometry of the interaction between CIP and the C3d fragment of C3 was investigated by size-exclusion chromatography. When C3d and CIP were loaded separately onto the column, 2 elution peaks corresponding to the 35 and 15 kDa monomeric forms of CIP and C3d, respectively, were detected. Coincubation of CIP with C3d yielded, instead, a single peak of ∼50 kDa, in agreement with the theoretical MW of the C3d–CIP complex, suggesting a binding ratio of 1:1 between the 2 proteins (**Fig. 4*A***).

SPR experiments, in which purified C3d was immobilized onto the surface of a dextran chip and CIP was added in concentrations ranging from 0.039 to 5 μM, confirmed a concentration-dependent interaction closely fitting to a Langmuir 1:1 kinetic model (Fig. 4*B*). The GBS protein bound to C3d with an apparent *K*_d_ of 79 ± 0.62 nM (*K*_on_, 1.08 ± 0.25 × 10^4^ M^−1^s^−1^; *K*_off_, 8.5 ± 0.46 × 10^−4^ s^−1^), in agreement with the affinity measured by ELISA.

**Figure 5:**
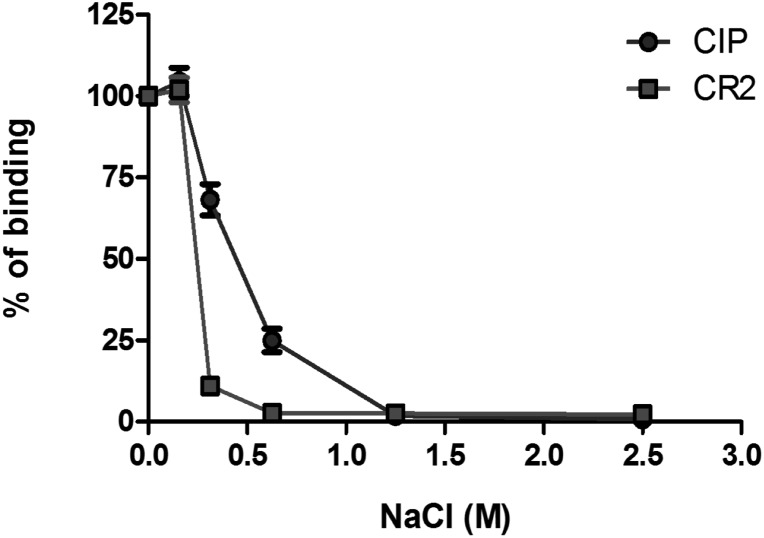
Ionic strength–dependent interaction of CIP, with C3d assessed by ELISA. Microtiter wells were coated with 250 ng of C3d. The wells were probed with CIP or CD21 diluted in a buffer containing increasing concentrations of NaCl (0–2.5 M). Complex formation was revealed through anti-CIP or anti-CD21 IgG pAb, followed by an HRP-conjugated secondary antibody.

CIP is a highly positively charged protein with 20 Lys and 8 Arg out of 153 aa residues and a calculated isoelectric point of 9.62. The CR2–CD21 receptor is also rich in positively charged residues, and its interaction with C3d is known to be ionic strength dependent (27). The effect of ionic strength on the interaction between CIP and C3d was investigated by ELISA. As shown in **Fig. 5**, binding of surface-coated C3d to both soluble CR2–CD21 and CIP was inhibited by increasing NaCl concentrations, suggesting an electrostatic contribution to CIP interaction with C3d.

Recent structural studies revealed that C3d-binding staphylococcal proteins are folded in a 3-helix-bundle conformation (19, 23, 28). The circular dichroism spectrum of CIP in the far-UV indicated a prevalence of an α-helix secondary structure (46%), with a maximum positive molar ellipticity at ∼7800° cm^2^/dmol, a maximum negative molar ellipticity at around −4500° cm^2^/dmol, and the presence of β sheets (36%) and random coil (18%) structures (Supplemental Fig. S1).

### Mapping of the CIP peptide involved in C3d binding

The C3d binding region of the CIP was investigated by HDx-MS. In this type of experiment, the interface between the binding partners can occlude solvent accessibility, thereby reducing the deuterium-exchange rate of the backbone amide hydrogens. After pepsin digestion of the protein of interest, the resulting peptides were compared for their masses. A consistent mass shift of 1 Da was considered the threshold for a significant exchange of 1 deuterium atom (28, 29).

**Figure 6:**
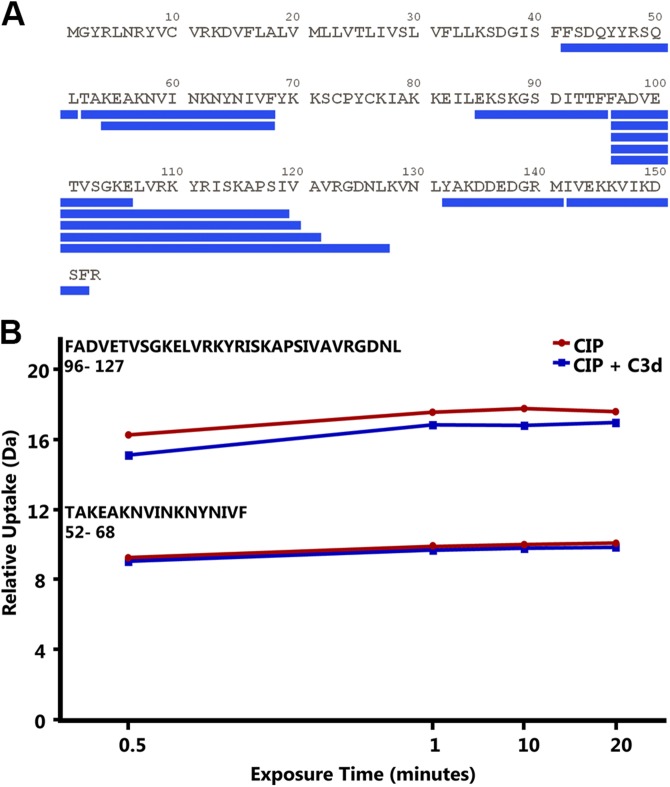
HDx-MS analysis of CIP interaction with C3d. *A*) Peptide coverage of the CIP protein: blue lines indicate the peptides recovered by pepsin treatment in the HDx experiment. *B*) Deuterium uptake over 20 min for the peptides 96–127 and 52–68 in the absence (red curve) or presence (blue curve) of C3d.

The CIP was incubated alone or in presence of C3d in a deuterated solution for different periods. Eleven CIP peptides, corresponding to 76% coverage of the mature protein, were generated from the subsequent pepsin digestion (**Fig. 6*A***). Deuterium incorporation in the recovered peptides was monitored by mass spectrometry. As shown in Fig. 6*B*, a consistent difference of 1 Da of deuterium uptake was detected for fragments 96–127. No differences in exchange ratio were detected for any of the remaining peptides, including those partially overlapping with the region 96–127, and starting from aa 96 up to 121. The data suggested that the CIP amino acids involved in the C3d interaction were located in the stretch between residues 122 and 127 that contains an Arg residue (R123).

### Soluble CIP interferes with the interaction between C3d and CR2/CD21

As previously reported, CIP inhibits the formation of the C4b2a classical and lectin C3 convertase, but not the C3bBb alternative pathway convertase (15).

We hypothesized that the interaction between CIP and C3d could interfere with the formation of the C3d–CR2/CD21 complex and reduce the kinetics of antibody-mediated B-cell intracellular signaling, as already observed for the staphylococcal Efb (22).

**Figure 7:**
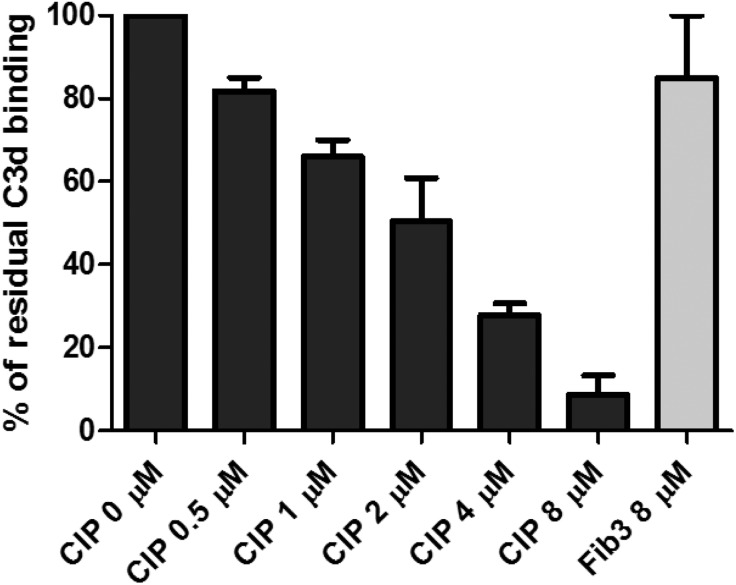
ELISA competitive experiments showing that CIP inhibits the formation of the C3d–CR2/CD21 complex. Microtiter wells were coated with 100 ng of CR2/CD21. The wells were probed with C3d preincubated with or without increasing concentrations of CIP or the highest concentration of Fib3 protein, followed by anti-C3 pAb and HRP-conjugated secondary antibody to detect the binding. Data are expressed as the percentage of the absorbance values detected in the absence of a competitor.

To investigate the capacity of CIP to interfere with the C3d–CR2/CD21 interaction, we performed a competitive ELISA experiment in which soluble C3d was preincubated with increasing concentrations of CIP, and the solution was overlaid on microtiter plates coated with CR2/CD21. Binding of C3d to the CR2/CD21 receptor was revealed with an anti-C3 pAb and secondary antibody incubation. The GBS protein Fib3 (25) was used as negative control. As shown in **Fig. 7**, CIP inhibited the C3d–CD21 interaction in a dose-dependent manner, whereas no effect was observed for Fib3 tested at the highest concentration.

### CIP inhibits the formation of the C3d–CR2/CD21 complex on B cells

**Figure 8:**
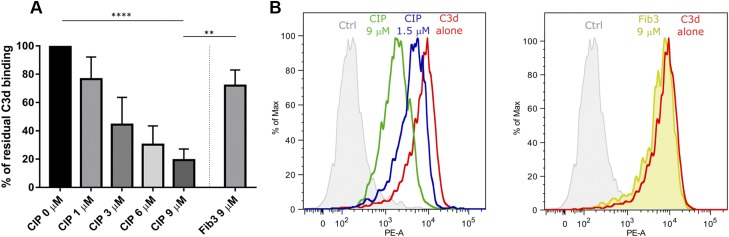
Flow cytometry analysis of C3d interaction with B cells in presence or absence of CIP. *A*) Biotinylated C3d was preincubated with SA and then with 1–9 μM of CIP, 9 μM of Fib3, or buffer alone. Each mixture was added to Raji B cells and treated as described. Binding of the biotinylated C3d–SA complex to cells was revealed by flow cytometry using a C3d-specific mAb and a phycoerythrin-labeled secondary antibody. Mean fluorescence intensity values of the peaks were analyzed by the FlowJo software. The graph shows the percentage of the residual mean fluorescence intensity values in the presence of a competitor compared with buffer alone, as derived from 4 independent experiments. *B*) Flow cytometry analysis of C3d binding to enriched B cells from human PBMCs in presence or absence of 1.5 or 9 μM CIP or 9 μM of Fib3; experimental conditions were the same as in *A*.

We subsequently investigated whether CIP could inhibit the interaction between soluble C3d and CR2/CD21 present on the B-cell surface. It has been previously reported that C3d multimers can bind to CR2/CD21 on the surface of the Raji-immortalized B-cell line (22, 30). Flow cytometry experiments confirmed binding of biotinylated C3d preincubated with SA (C3d–biotin–SA) to Raji cells. Increasing concentrations of CIP were preincubated with the C3d–biotin–SA complex before adding the mixture to the cells, followed by flow cytometry analysis. As shown in **Fig. 8*A***, the presence of CIP interfered with C3d binding to the B cells in a dose-dependent manner. Conversely, the same inhibitory effect was not detected when using the highest concentration of the negative control Fib3. CIP inhibition of C3d binding was also confirmed in a similar experiment with B cells enriched from human PBMCs, as evidenced by a dose-dependent shift in fluorescence in the corresponding flow cytometry histograms (Fig. 8*B*).

### CIP inhibits B-cell intracellular signaling

**Figure 9:**
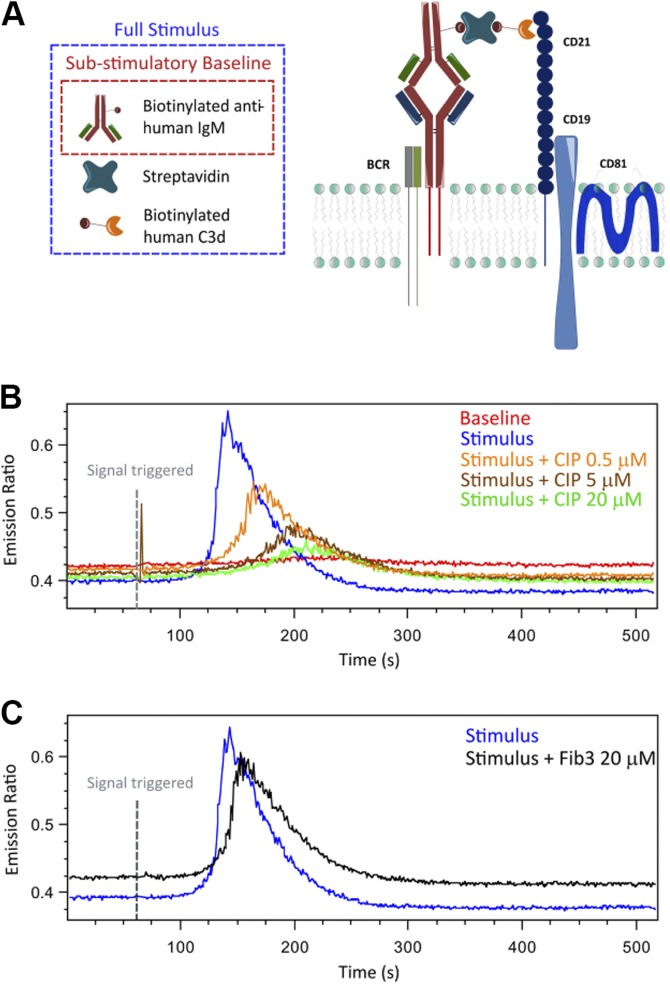
Flow cytometry analysis of B-cell intracellular-calcium mobilization in presence or absence of CIP. *A*) Diagram representation of the stimulus that triggers intracellular calcium increase (blue box); biotinylated anti-human IgM antibody (red box) is used to measure the substimulatory baseline before each experiment. The stimulus is designed to crosslink the B-cell receptor (BCR) to CR2/CD21. *B*, *C*) Raji cells were preincubated with Fura Red, followed by the stimulus (see *A*). Either 0.5–20 μM of CIP (*B*) or 20 μM of Fib3 (*C*) were preincubated with C3d–biotin–SA to assess their inhibition effects; changes in cellular fluorescence were monitored during 540 s and analyzed by flow cytometry. One representative of 3 experiments performed is shown.

The implications of CIP–C3d interaction on B-cell intracellular signaling were investigated in experiments in which C3d–biotin–SA and biotinylated anti-human IgM (BioLegend, San Diego, CA, USA) were combined to trigger intracellular calcium increase, as depicted in the diagram shown in **Fig. 9*A***. Before the addition of stimuli, Raji B cells were treated with Fura Red, a cell-permeant fluorophore that changes its emission wavelength when bound to calcium. Changes in cellular fluorescence at different times were recorded by flow cytometry, and the acquisition of fluorescent cells, incubated with biotin–anti-human IgM only, was used as substimulatory baseline. To assess the CIP inhibition effect, intracellular signaling was triggered in the absence or in presence of increasing concentrations of the protein or in the presence of the highest concentration of Fib3 as a negative control. As shown in Fig. 9*B*, CIP decreased intracellular calcium release in a dose-dependent manner with maximum inhibition at 20 μM of CIP. Conversely, fluorescence was not affected by the presence of Fib3 (Fig. 9*C*).

## DISCUSSION

The GBS CIP protein was first identified as a virulence factor secreted by GBS, showing a capacity to bind the complement proteins C4 and C4b and to block the deposition of C3b on the bacterial surface *via* the lectin and classical pathways (15). The present study identified, as new ligands for CIP, the C3 central complement component and its thioester containing effector fragments C3b and C3d and revealed new complement evasion mechanisms for this GBS protein.

By sequence homology comparisons with staphylococcal-secreted virulence factors, CIP appeared as a protein chimera showing partial similarity to the C4-binding protein Eap from *S. aureus* in its N-terminal region and to the C3b/C3d binding proteins Efb and Sbi in their C-terminal regions (15) (Supplemental Fig. S2). The CIP C4 and C3 binding sites appeared to be independent, based on competition experiments.

The interaction between Efb or Sbi and C3 induces conformational changes in C3b that abolish the C3bBb convertase function blocking downstream-activation events (20, 28). Moreover, Sbi was shown to form a tripartite complex with C3b and factor H that contributes to its inhibitory effect toward the complement alternative pathway (20). A similar role of the GBS CIP protein in preventing the formation/activation of the C3bBb convertase could have been expected based on its C3b-binding capacity but was excluded in our previous investigations (15). The molecular basis for the different modulatory capacity toward the alternative pathway between CIP and the Efb and Sbi staphylococcal proteins deserves further investigations. Here, we focused our attention on the interaction with C3d, the last degradation fragment of C3, which possibly contains the complete CIP binding site. A saturable, univalent interaction between CIP and C3d was demonstrated by coupling ligand and receptor in solid phase supports and by size-exclusion chromatography. SPR experiments confirmed an intrinsic affinity between CIP and C3d in the order of 0.1 μM, slightly higher than the *K*_d_ measured for the staphylococcal Sbi (23) and less than the one reported for Efb (31).

Besides having a key role in innate immune surveillance, the complement system is implicated in the engagement of adaptive responses (32), and several *in vivo* studies support a role for CRs in the acquisition of target antigens (33). In particular, the C3d/C3dg physiologic degradation products of C3 enhance B-cell signaling by simultaneously binding the antigen–B-cell receptor complex and the coreceptor complex formed by CR2/CD21, CD19, and CD81. This receptor coligation has a profound molecular adjuvant effect that lowers the threshold of antigen required for B-cell activation by >1000-fold (34). The C3d-dependent crosslink also directs B cells toward their T-cell boundary, where they further differentiate through clonal expansion, somatic hypermutation, affinity maturation, and Ig class-switch recombination (35). Furthermore, the retention of C3d-opsonized antigens through CR2/CD21 on follicular dendritic cells in the lymph node is essential for the generation of high-affinity antibodies and memory B cells (36).

Here, we show that binding of CIP to C3d prevents the interaction of this protein with the CR2/CD21 B-cell receptor both *in vitro* and *ex vivo* and that this interference effect results in decreased intracellular signaling, as measured by lower levels of calcium release. Therefore, the data point toward a new role for the CIP protein in counteracting adaptive immunity by modulating the activity of the C3d complement effector and may represent a new example of the coevolution between the GBS microorganism and its human host.

The CIP concentrations used in our experiments were in the range of ∼0.1–10 µM, and equimolar amounts of CIP and C3d were used for analyzing the interaction between the 2 proteins by size-exclusion chromatography. An approximate quantification of CIP by dot blot in the supernatant of a GBS neonatal strain grown *in vitro* to stationary phase in parallel to recombinant CIP, indicated concentrations of ∼0.3–0.5 µM (data not shown). This value could represent an underestimation because CIP is able to bind to the bacterial surface (15) not allowing a reliable quantification of the secreted form. We previously observed that CIP was differentially expressed in different growth media, and its expression might also be regulated during infection, making it difficult to draw definite conclusions on the physiologic relevance of concentrations measured from an *in vitro* culture.

Deep genomics studies have indicated that lateral transfer and recombination are strongly implicated in the evolution of GBS. In particular, phage insertions generate interstrain diversity and provide the pathogen with a number of virulence factors that facilitate its survival in the host (37). Of note, the gene coding for CIP is located in a hot spot phage insertion region of ∼20 kbp (15), which also contains the *bspC* locus implicated in biofilm formation (38, 39). Interestingly, group A *Streptococcus* isolates that belong to serotypes associated with maternal–fetal urogenital infections contain a similar phage insertion that also encodes a secreted protein 46% identical to CIP (15), suggesting interspecies lateral transfer.

Cocrystal structural analysis highlighted essential salt bridges and hydrogen bond interactions between a concave acidic pocket from C3d and positively charged residues in the surface patches of CR2/CD21 SCR (short consensus repeat) 1 and 2 domains (27, 40). A similar type of interaction between C3d and cation residues present on α helix 2 of the staphylococcal Efb and Sbi was demonstrated by cocrystallization and mutagenesis experiments (23, 28). Secondary structure predictions confirmed by circular dichroism analysis indicate that the C-terminal region of CIP also presents an α-helical structure. Interestingly, HDx-MS led to the identification of a CIP peptide interacting with C3d that contains an arginine (R123) separated by 6 residues from an asparagine (N130). The 2 residues perfectly matched the R131 and N138 present on α helix 2 of the staphylococcal Efb/Efb-C and the R231 and N238 of Sbi. Unfortunately, the CIP peptide containing N130 was not recovered by MS possibly because of the proximity of pepsin-sensitive residues. Two recombinant variants of CIP, the first containing a substitution of R123 with an alanine (A) residue and the second a double-mutant R123 to A plus N130 to A, were expressed in *E. coli* to experimentally confirm their role in C3d binding. Despite high expression levels, the 2 mutant proteins were poorly soluble in aqueous buffer and quickly precipitated. Future cocrystallization experiments will be instrumental to confirm the amino acid residues directly involved in the interactions between CIP and C3d.

The gastrointestinal and urogenital GBS colonization sites are rich in mucosal-associated lymphoid tissues containing many B cells, in which CIP could exert its immunomodulation effect (41).

In addition to having an important role as the ligand of CR2/CD21, C3d also binds the CR3 (CD11b/CD18) on dendritic cells and macrophages *via* its integrin I domain (42). A possible additional effect of CIP not investigated here could be the prevention of the interaction between C3d and CR3 on antigen-presenting cells, limiting the transport of GBS antigens to the lymph nodes and their presentation to resting B cells.

To investigate the effect of CIP on GBS virulence *in vivo*, we tried to obtain an isogenic *cip* mutant in a GBS neonatal strain but were unsuccessful. Previous experiments had shown that GBS survival in human blood was enhanced after preincubation of the bacteria with recombinant CIP (15). It is tempting to speculate that the observed antiphagocytic effect of CIP could rely on its capacity to bind both C4b and C3. Indeed, in addition to modulating the formation of C3b *via* the classical/lectin pathways, as previously reported, the CIP protein could prevent binding of complement-labeled bacteria to CR3 and CR1/CD35 receptors in neutrophils.

Finally, it has been postulated that differentiation of autoreactive B cells could also involve coreceptor engagement of C3d along with B-cell receptor binding to self-antigens (32). If that hypothesis is confirmed, proteins capable of interfering with the C3d–CR2/CD21 interaction, such as CIP or their derived peptides, could represent valuable therapeutic tools for the modulation of aberrant B-cell responses to combat antibody-mediated autoimmune disorders.

## Supplementary Material

This article includes supplemental data. Please visit *http://www.fasebj.org* to obtain this information.

## Abbreviations

bC3d: biotinylated C3d
CD: cluster of differentiation
CIP: complement interfering protein
CR1/2/3: complement receptor 1/2/3
Eap: extracellular adherence protein
Efb: extracellular fibrinogen-binding protein
Ehp: extracellular fibrinogen-binding homologous protein
Fib3: fibrinogen-binding protein 3
GBS: group B *Streptococcus*
HDx-MS: hydrogen deuterium exchange–mass spectrometry
HRP: horseradish peroxidase
PBMC: peripheral blood mononuclear cell
PBST: PBS supplemented with 0.1% (v/v) Tween 20
SA: streptavidin
Sbi: staphylococcal-binding IgG
SHT: streptococcal histidine triad
SPR: surface plasmon resonance

## References

[1] Gibbs R. S., Schrag S., Schuchat A. (2004) Perinatal infections due to group B streptococci. Obstet. Gynecol. 104, 1062–1076 15516403 10.1097/01.AOG.0000144128.03913.c2

[2] Herbert M. A., Beveridge C. J. E., Saunders N. J. (2004) Bacterial virulence factors in neonatal sepsis: group B streptococcus. Curr. Opin. Infect. Dis. 17, 225–229 15166825 10.1097/00001432-200406000-00009

[3] Heath P. T., Schuchat A. (2007) Perinatal group B streptococcal disease. Best Pract. Res. Clin. Obstet. Gynaecol. 21, 411–424 17336588 10.1016/j.bpobgyn.2007.01.003

[4] Fairlie T., Zell E. R., Schrag S. (2013) Effectiveness of intrapartum antibiotic prophylaxis for prevention of early-onset group B streptococcal disease. Obstet. Gynecol. 121, 570–577 23635620 10.1097/AOG.0b013e318280d4f6

[5] Berends E. T., Kuipers A., Ravesloot M. M., Urbanus R. T., Rooijakkers S. H. (2014) Bacteria under stress by complement and coagulation. FEMS Microbiol. Rev. 38, 1146–1171 25065463 10.1111/1574-6976.12080

[6] Lindahl G., Stålhammar-Carlemalm M., Areschoug T. (2005) Surface proteins of *Streptococcus agalactiae* and related proteins in other bacterial pathogens. Clin. Microbiol. Rev. 18, 102–127 15653821 10.1128/CMR.18.1.102-127.2005 PMC544178

[7] Carlin A. F., Lewis A. L., Varki A., Nizet V. (2007) Group B streptococcal capsular sialic acids interact with siglecs (immunoglobulin-like lectins) on human leukocytes. J. Bacteriol. 189, 1231–1237 16997964 10.1128/JB.01155-06 PMC1797352

[8] Carlin A. F., Chang Y. C., Areschoug T., Lindahl G., Hurtado-Ziola N., King C. C., Varki A., Nizet V. (2009) Group B *Streptococcus* suppression of phagocyte functions by protein-mediated engagement of human Siglec-5. J. Exp. Med. 206, 1691–1699 19596804 10.1084/jem.20090691 PMC2722167

[9] Marques M. B., Kasper D. L., Pangburn M. K., Wessels M. R. (1992) Prevention of C3 deposition by capsular polysaccharide is a virulence mechanism of type III group B streptococci. Infect. Immun. 60, 3986–3993 1398910 10.1128/iai.60.10.3986-3993.1992 PMC257427

[10] Jarva H., Hellwage J., Jokiranta T. S., Lehtinen M. J., Zipfel P. F., Meri S. (2004) The group B streptococcal β and pneumococcal Hic proteins are structurally related immune evasion molecules that bind the complement inhibitor factor H in an analogous fashion. J. Immunol. 172, 3111–3118 14978117 10.4049/jimmunol.172.5.3111

[11] Maruvada R., Prasadarao N. V., Rubens C. E. (2009) Acquisition of factor H by a novel surface protein on group B *Streptococcus* promotes complement degradation. FASEB J. 23, 3967–3977 19608625 10.1096/fj.09-138149 PMC2775014

[12] Santi I., Scarselli M., Mariani M., Pezzicoli A., Masignani V., Taddei A., Grandi G., Telford J. L., Soriani M. (2007) BibA: a novel immunogenic bacterial adhesin contributing to group B *Streptococcus* survival in human blood. Mol. Microbiol. 63, 754–767 17212592 10.1111/j.1365-2958.2006.05555.x

[13] Cheng Q., Debol S., Lam H., Eby R., Edwards L., Matsuka Y., Olmsted S. B., Cleary P. P. (2002) Immunization with C5a peptidase or peptidase-type III polysaccharide conjugate vaccines enhances clearance of group B streptococci from lungs of infected mice. Infect. Immun. 70, 6409–6415 12379721 10.1128/IAI.70.11.6409-6415.2002 PMC130386

[14] Brown C. K., Gu Z. Y., Matsuka Y. V., Purushothaman S. S., Winter L. A., Cleary P. P., Olmsted S. B., Ohlendorf D. H., Earhart C. A. (2005) Structure of the streptococcal cell wall C5a peptidase. Proc. Natl. Acad. Sci. USA 102, 18391–18396 16344483 10.1073/pnas.0504954102 PMC1317908

[15] Pietrocola G., Rindi S., Rosini R., Buccato S., Speziale P., Margarit I. (2016) The group B *Streptococcus*-secreted protein CIP interacts with C4, preventing C3b deposition via the lectin and classical complement pathways. J. Immunol. 196, 385–394 26608922 10.4049/jimmunol.1501954 PMC4683360

[16] Woehl J. L., Stapels D. A. C., Garcia B. L., Ramyar K. X., Keightley A., Ruyken M., Syriga M., Sfyroera G., Weber A. B., Zolkiewski M., Ricklin D., Lambris J. D., Rooijakkers S. H. M., Geisbrecht B. V. (2014) The extracellular adherence protein from *Staphylococcus aureus* inhibits the classical and lectin pathways of complement by blocking formation of the C3 proconvertase. J. Immunol. 193, 6161–6171 25381436 10.4049/jimmunol.1401600 PMC4258549

[17] Woehl J. L., Ramyar K. X., Katz B. B., Walker J. K., Geisbrecht B. V. (2017) The structural basis for inhibition of the classical and lectin complement pathways by *S. aureus* extracellular adherence protein. Protein Sci. 26, 1595–1608 28512867 10.1002/pro.3195 PMC5521547

[18] Lee L. Y. L., Höök M., Haviland D., Wetsel R. A., Yonter E. O., Syribeys P., Vernachio J., Brown E. L. (2004) Inhibition of complement activation by a secreted *Staphylococcus aureus* protein. J. Infect. Dis. 190, 571–579 15243934 10.1086/422259

[19] Hammel M., Sfyroera G., Pyrpassopoulos S., Ricklin D., Ramyar K. X., Pop M., Jin Z., Lambris J. D., Geisbrecht B. V. (2007) Characterization of Ehp, a secreted complement inhibitory protein from *Staphylococcus aureus*. J. Biol. Chem. 282, 30051–30061 17699522 10.1074/jbc.M704247200

[20] Haupt K., Reuter M., van den Elsen J., Burman J., Hälbich S., Richter J., Skerka C., Zipfel P. F. (2008) The *Staphylococcus aureus* protein Sbi acts as a complement inhibitor and forms a tripartite complex with host complement factor H and C3b. PLoS Pathog. 4, e1000250 19112495 10.1371/journal.ppat.1000250 PMC2602735

[21] Burman J. D., Leung E., Atkins K. L., O’Seaghdha M. N., Lango L., Bernadó P., Bagby S., Svergun D. I., Foster T. J., Isenman D. E., van den Elsen J. M. (2008) Interaction of human complement with Sbi, a staphylococcal immunoglobulin-binding protein: indications of a novel mechanism of complement evasion by *Staphylococcus aureus*. J. Biol. Chem. 283, 17579–17593 18434316 10.1074/jbc.M800265200 PMC2649420

[22] Ricklin D., Ricklin-Lichtsteiner S. K., Markiewski M. M., Geisbrecht B. V., Lambris J. D. (2008) Cutting edge: members of the *Staphylococcus aureus* extracellular fibrinogen-binding protein family inhibit the interaction of C3d with complement receptor 2. J. Immunol. 181, 7463–7467 19017934 10.4049/jimmunol.181.11.7463 PMC2673544

[23] Upadhyay A., Burman J. D., Clark E. A., Leung E., Isenman D. E., van den Elsen J. M. H., Bagby S. (2008) Structure-function analysis of the C3 binding region of *Staphylococcus aureus* immune subversion protein Sbi. J. Biol. Chem. 283, 22113–22120 18550524 10.1074/jbc.M802636200 PMC2494919

[24] Cherukuri A., Cheng P. C., Pierce S. K. (2001) The role of the CD19/CD21 complex in B cell processing and presentation of complement-tagged antigens. J. Immunol. 167, 163–172 11418645 10.4049/jimmunol.167.1.163

[25] Margarit I., Bonacci S., Pietrocola G., Rindi S., Ghezzo C., Bombaci M., Nardi-Dei V., Grifantini R., Speziale P., Grandi G. (2009) Capturing host-pathogen interactions by protein microarrays: identification of novel streptococcal proteins binding to human fibronectin, fibrinogen, and C4BP. FASEB J. 23, 3100–3112 19417080 10.1096/fj.09-131458

[26] Domina M., Lanza Cariccio V., Benfatto S., Venza M., Venza I., Donnarumma D., Bartolini E., Borgogni E., Bruttini M., Santini L., Midiri A., Galbo R., Romeo L., Patanè F., Biondo C., Norais N., Masignani V., Teti G., Felici F., Beninati C. (2016) Epitope mapping of a monoclonal antibody directed against neisserial heparin binding antigen using next generation sequencing of antigen-specific libraries. PLoS One 11, e0160702 27508302 10.1371/journal.pone.0160702 PMC4980009

[27] Van den Elsen J. M. H., Isenman D. E. (2011) A crystal structure of the complex between human complement receptor 2 and its ligand C3d. Science 332, 608–611 21527715 10.1126/science.1201954

[28] Hammel M., Sfyroera G., Ricklin D., Magotti P., Lambris J. D., Geisbrecht B. V. (2007) A structural basis for complement inhibition by *Staphylococcus aureus.* Nat. Immunol. 8, 430–437 17351618 10.1038/ni1450

[29] Malito E., Faleri A., Lo Surdo P., Veggi D., Maruggi G., Grassi E., Cartocci E., Bertoldi I., Genovese A., Santini L., Romagnoli G., Borgogni E., Brier S., Lo Passo C., Domina M., Castellino F., Felici F., van der Veen S., Johnson S., Lea S. M., Tang C. M., Pizza M., Savino S., Norais N., Rappuoli R., Bottomley M. J., Masignani V. (2013) Defining a protective epitope on factor H binding protein, a key meningococcal virulence factor and vaccine antigen. Proc. Natl. Acad. Sci. USA 110, 3304–3309 23396847 10.1073/pnas.1222845110 PMC3587270

[30] Buhlmann D., Eberhardt H. U., Medyukhina A., Prodinger W. M., Figge M. T., Zipfel P. F., Skerka C. (2016) FHR3 blocks C3d-mediated coactivation of human B cells. J. Immunol. 197, 620–629 27279373 10.4049/jimmunol.1600053

[31] Haspel N., Ricklin D., Geisbrecht B. V., Kavraki L. E., Lambris J. D. (2008) Electrostatic contributions drive the interaction between *Staphylococcus aureus* protein Efb-C and its complement target C3d. Protein Sci. 17, 1894–1906 18687868 10.1110/ps.036624.108 PMC2578803

[32] Carroll M. C., Isenman D. E. (2012) Regulation of humoral immunity by complement. Immunity 37, 199–207 22921118 10.1016/j.immuni.2012.08.002 PMC5784422

[33] Gonzalez S. F., Degn S. E., Pitcher L. A., Woodruff M., Heesters B. A., Carroll M. C. (2011) Trafficking of B cell antigen in lymph nodes. Annu. Rev. Immunol. 29, 215–233 21219172 10.1146/annurev-immunol-031210-101255

[34] Dempsey P. W., Allison M. E., Akkaraju S., Goodnow C. C., Fearon D. T. (1996) C3d of complement as a molecular adjuvant: bridging innate and acquired immunity. Science 271, 348–350 8553069 10.1126/science.271.5247.348

[35] Cyster J. G., Ansel K. M., Reif K., Ekland E. H., Hyman P. L., Tang H. L., Luther S. A., Ngo V. N. (2000) Follicular stromal cells and lymphocyte homing to follicles. Immunol. Rev. 176, 181–193 11043777 10.1034/j.1600-065x.2000.00618.x

[36] Roozendaal R., Carroll M. C. (2007) Complement receptors CD21 and CD35 in humoral immunity. Immunol. Rev. 219, 157–166 17850488 10.1111/j.1600-065X.2007.00556.x

[37] Glaser P., Rusniok C., Buchrieser C., Chevalier F., Frangeul L., Msadek T., Zouine M., Couvé E., Lalioui L., Poyart C., Trieu-Cuot P., Kunst F. (2002) Genome sequence of *Streptococcus agalactiae*, a pathogen causing invasive neonatal disease. Mol. Microbiol. 45, 1499–1513 12354221 10.1046/j.1365-2958.2002.03126.x

[38] Chuzeville S., Dramsi S., Madec J. Y., Haenni M., Payot S. (2015) Antigen I/II encoded by integrative and conjugative elements of *Streptococcus agalactiae* and role in biofilm formation. Microb. Pathog. 88, 1–9 26232503 10.1016/j.micpath.2015.07.018

[39] Rego S., Heal T. J., Pidwill G. R., Till M., Robson A., Lamont R. J., Sessions R. B., Jenkinson H. F., Race P. R., Nobbs A. H. (2016) Structural and functional analysis of cell wall-anchored polypeptide adhesin BspA in *Streptococcus agalactiae*. J. Biol. Chem. 291, 15985–16000 27311712 10.1074/jbc.M116.726562 PMC4965550

[40] Nagar B., Jones R. G., Diefenbach R. J., Isenman D. E., Rini J. M. (1998) X-ray crystal structure of C3d: a C3 fragment and ligand for complement receptor 2. Science 280, 1277–1281 9596584 10.1126/science.280.5367.1277

[41] Suzuki K., Grigorova I., Phan T. G., Kelly L. M., Cyster J. G. (2009) Visualizing B cell capture of cognate antigen from follicular dendritic cells. J. Exp. Med. 206, 1485–1493 19506051 10.1084/jem.20090209 PMC2715076

[42] Bajic G., Yatime L., Sim R. B., Vorup-Jensen T., Andersen G. R. (2013) Structural insight on the recognition of surface-bound opsonins by the integrin I domain of complement receptor 3. Proc. Natl. Acad. Sci. USA 110, 16426–16431 24065820 10.1073/pnas.1311261110 PMC3799375

