# Supplementary material for: The Streptococcus agalactiae complement interfering protein combines multiple complement-inhibitory mechanisms by interacting with both C4 and C3 ligands
Source: FASEB J. 2018 Dec 19;33(3):4448–57. doi: 10.1096/fj.201801991R (PMC6404586; doi:10.1096/fj.201801991R)
Supplement: Supplementary file 1 [file fj.201801991R.sf1.docx]

**SUPPLEMENTARY FIGURES**

**Supplementary Figure 1. CIP secondary structure prediction through Circular Dichroism analysis.** Far-UV CD spectra of CIP was recorded at 20 °C in 20 mM phosphate buffer at pH 7.0 at 0.1 mg/ml protein concentration. The reported spectra are the average of five scans, corrected for buffer blank, and were smoothed.


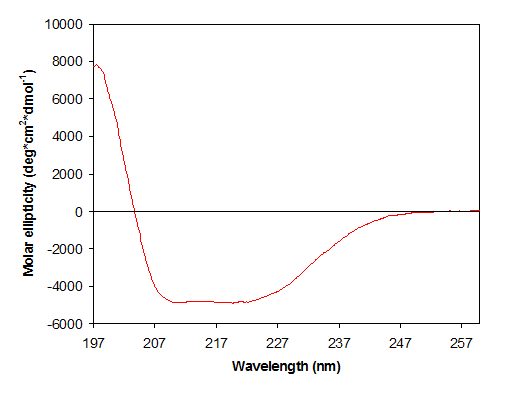


**Supplementary Figure 2**. **Sequence alignment among the C-terminal regions of three bacterial C3d-binding proteins.** Partial amino acid sequence comparison between Efb-C, Sbi-IV (from *S. aureus* USA300 and NCTC 8325 strains respectively) and CIP (from GBS COH1). Identical amino acid residues among the three proteins are indicated in yellow; identical amino acid residues shared by Efb-C and CIP are in green, by Sbi and CIP in light blue and by Efb-C and Sbi in gray; red amino acid residues outline the key residues for C3d binding on Efb-C and Sbi, while the red numbers indicate their position The alpha helices of Efb-C and Sbi are indicated by the blue lines; the green line on CIP covers the fragments detected in the HDx experiment. The alignment was performed by Geneious software.

**
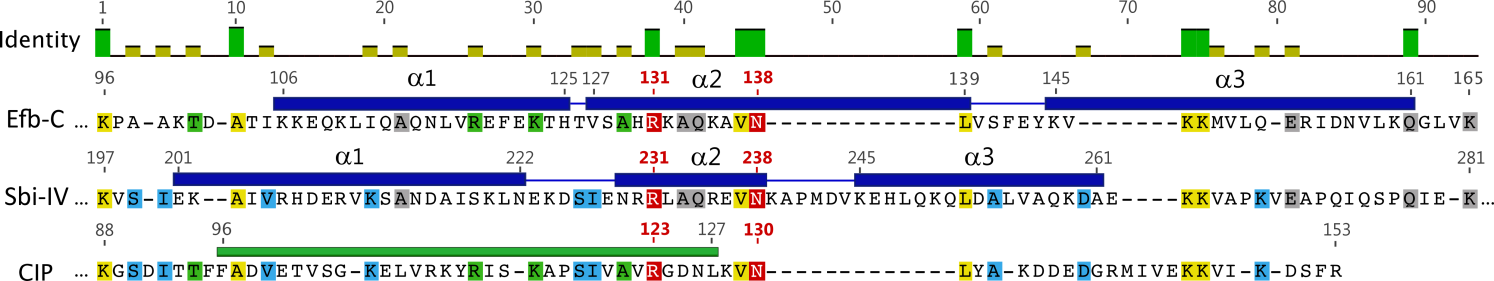
**
